# Control of expression of the ICE R391 encoded UV-inducible cell-sensitising function

**DOI:** 10.1186/1471-2180-13-195

**Published:** 2013-08-29

**Authors:** Patricia Armshaw, J Tony Pembroke

**Affiliations:** 1 Molecular and Structural Biochemistry Laboratory, Department of Chemical and Environmental Sciences, University of Limerick, Limerick, Ireland

**Keywords:** SXT/R391 ICE, Type IV secretion system, Conjugative transfer, UV-induced sensitisation and cytotoxicity

## Abstract

**Background:**

Many SXT/R391-like enterobacterial Integrative Conjugative Elements (ICEs) have been found to express an atypical, *recA*-dependent, UV-inducible, cell-sensitising phenotype observed as a reduction in post-irradiation cell survival rates in host cells. Characterisation of a complete deletion library of the prototype ICE R391 identified the involvement of three core ICE genes, *orfs90*/*91* encoding a putative transcriptional enhancer complex, and *orf43*, encoding a putative type IV secretion system, outer membrane-associated, conjugative transfer protein.

**Results:**

In this study, expression analysis of *orf43* indicated that it was up-regulated as a result of UV irradiation in an *orfs90/91-*dependent manner. Induced expression was found to be controlled from a site preceding the gene which required functional *orfs90/91*. Expression of *orfs90/91* was in turn found to be regulated by *orf96*, a λ cI-like regulator. Targeted construction of ICE R391 deletions, RT-PCR and qRT-PCR analysis confirmed a regulatory link between *orfs90/91* and *orf43* while site-directed mutagenesis of *orf43* suggested an association with the cell membrane was a prerequisite for the cytotoxic effect.

**Conclusions:**

Because of the *recA*-dependence of the effect, we hypothesise that UV induction of RecA results in cleavage of the cI-like ICE-encoded repressor protein, the product of *orf96*. This in turn allows expression of the transcriptional enhancer complex encoded by *orfs90/91*, which we conclude stimulates transcription of *orf43,* whose product is directly responsible for the effect.

## Background

Integrative Conjugative Elements (ICEs) are a class of bacterial mobile genetic elements that encode features necessary for their site-specific integration and excision from host genomes, self-circularisation and transfer by conjugation [1,2]. ICEs are divided into families based on similarity between core genes (specifically the integrase gene) and the site of integration they utilise within host chromosomes. The SXT/R391 family share a highly similar integrase gene and integrate into the *prfC* gene of enterobacterial hosts [1,3]. In addition to encoding host beneficial traits such as antibiotic resistance determinants [3-5], many SXT/R391 family ICEs express an unusual cell-sensitising function [6-8]. Preliminary characterisation of the UV-inducible, cell-sensitising function of the prototype, ICE R391, determined the effect to be *recA*-dependent [6], while further analysis based on construction of a deletion library of ICE R391 found that three core ICE genes, namely *orfs90/91* and *orf43* were involved [8]. Deletion analysis also revealed that *orf96*, which encodes a putative λ cI-like repressor protein [9], could only be deleted in strains where *orfs90/91* had previously been removed suggesting that the repressor protein may prevent lethal expression of *orfs90/91*. Additionally, cloning and controlled expression of both *orfs90/91* and *orf43* revealed that expression of *orf43* alone was cytotoxic to wild type *E. coli* while expression of *orfs90/91* was only cytotoxic to wild type *E. coli* cells harbouring the ICE R391. This indicated that *orf43* was responsible for the observed UV-inducible cytotoxicity [8].

**Figure 1:**
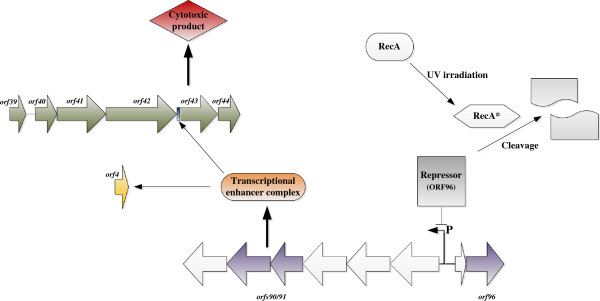
**Proposed induction pathway for the UV-inducible cell-sensitising function of ICE R391.** Stimulation of RecA to its active form (RecA*) by UV irradiation results in the cleavage of the putative *orfs90/91* repressor protein (*orf96*) allowing the transcription of *orfs90/91* which putatively encode a transcriptional enhancer complex that activates/increases expression of the *orf43* gene product as well as the previously documented UV-inducible *orf4* (*jef*) [14]. Expression of *orf43* is then cytotoxic to *E. coli* host cells. Evidence to support this hypothesised pathway includes: RecA has been well documented to be stimulated to its active form (RecA*) by single-stranded DNA generated from exposure to UV irradiation [16], the observation that the cell-sensitising function of ICE R391 requires the presence of *recA* in the host genome [6], the deletion of *orf96* encoding a putative repressor protein cannot be achieved without the previous deletion of *orfs90/91*[8], and *orfs90/91* have previously been documented to enhance the transcription of other ICE R391 genes after host cell exposure to UV irradiation, specifically *orf4* (*jef*), proposed to promote element excision from the host genome [14]. Additionally the ICE SXT homologs *setR* (*orf96*) and *setC/D* (*orfs90/91*) have been documented to have a similar *recA*-dependent, stress-inducible relationship [17].

RecA is a well-documented regulatory protein involved in UV-induced proteolysis of repressor proteins associated with the SOS response [10]. Induction of RecA (some 50 fold) following UV irradiation, results in cleavage of phage λ and phage λ -like cI repressors resulting in phage induction and indeed cleavage of other SOS repressors [10-13]. Bioinformatic analysis of the ICE R391 encoded *orf96* has shown it encodes a cI-like repressor protein with homology to phage λ^434^ cI [9], while analysis of the ICE R391-encoded *orfs90/91* has indicated that these genes may act as a putative transcriptional enhancer complex. It has been demonstrated that *orfs90/91* stimulate the expression of ICE specific genes such as *orf4* (*jef*, Figure 1) [14], which is an element-encoded excisionase, resulting in formation of increased levels of a circular form of the ICE, presumably as a transfer intermediate. The ICE R391-encoded *orf43*, has previously been found to be essential for ICE R391 transfer [8]. Indeed, based on bioinformatic homology, *orf43* is predicted to encode a putative TraV homolog, an outer membrane protein involved in the ICE type IV secretion system and thought to function in the construction and stabilisation of the outer-membrane portion of the mating pore required for ICE transfer by conjugation [15]. Deletion of the ICE R391-encoded *orf43* was recently shown to abolish the UV-inducible sensitising effect of this ICE while clones expressing *orf43* under arabinose control were shown to compliment for the transfer deficiency but additionally mimic the cell toxicity associated with UV induction [8].

Here, a model is proposed (Figure 1) for the control of this unusual ICE R391 UV-inducible sensitising effect based on expression data examining the key genes involved and supported by a number of directed ICE R391 deletions.

## Results and discussion

### *orfs90/91* stimulate *orf43* transcription after exposure to UV irradiation

We previously demonstrated that over-expression of *orf43* when cloned into the arabinose inducible pBAD33-*orf43* construct was responsible for the UV-inducible sensitisation observed in ICE R391 and other ICEs of the SXT/R391 family [8]. Mutagenesis data also suggested that the putative transcriptional controller encoded by *orfs90/91* was also involved, although not directly. To investigate the relationship between *orfs90/91* and *orf43*, we utilised both qualitative and quantitative RT-PCR targeting these genes in different mutant backgrounds and with and without UV irradiation.

**Figure 2:**
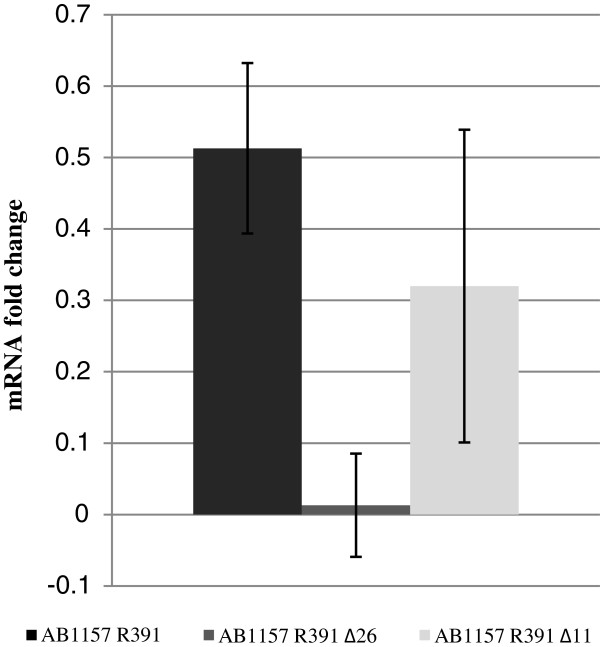
**Increase in *orf43 *mRNA levels after exposure to 40 J.m**^**-2 **^**UV irradiation.** Backgrounds analysed were AB1157 R391, AB1157 R391 ∆26 (∆*orfs90/91*) and AB1157 R391 ∆11 (∆*orfs40/41*). All results were normalised using the endogenous constitutively expressed *proC* gene. Average values were calculated from a minimum of 9 replicates for each strain analysed.

**Figure 3:**
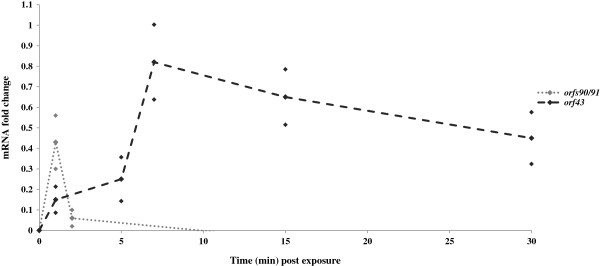
**Decay of AB1157 R391 *orfs90/91 *and *orf43 *mRNA levels after exposure to 40 J.m**^**-2 **^**UV irradiation.** All results were normalised using the endogenous constitutively expressed *proC* gene. Standard deviation is denoted by markers above and below all data points. *orfs90/91* specific mRNA levels were up-regulated immediately post exposure to UV irradiation but quickly decayed. *orf43* specific mRNA levels were maximally up-regulated 7 minutes post exposure and elevated levels were sustained for over 30 minutes post exposure.

qRT-PCR analysis of expression levels pre and post exposure to 40 J.m^-2^ UV-irradiation indicated that *orfs90/91*, *orf43* and the previously documented UV-inducible *orf4* (*jef*, Figure 1) [14] were up-regulated after exposure to UV irradiation. Analysis indicated that *orf4* (*jef*) specific mRNA levels were up-regulated 0.78 fold, *orf43*, 0.513 fold and *orfs9091*, 0.339 fold. In contrast other ICE R391 genes not involved in cell sensitisation [8] were not up-regulated post exposure: *aph* (encoding Kanamycin resistance) was down-regulated 0.23 fold post-exposure while *orf31* (encoding a putative Lon protease) was also down-regulated 0.19 fold post-exposure. Analysis of the up-regulated genes in mutant backgrounds indicated that in a Δ*orfs90/91* (∆26) background, *orf43* up-regulation was abolished (Figure 2) while analysis of *orfs90/91* transcription in a Δ*orf43* (∆14) background did not prevent *orfs90/91* specific mRNA up-regulation following UV irradiation (*orfs90/91* up-regulated 0.61 fold in AB1157 R391 ∆14). This indicated a dependency on *orfs90/91* for *orf43* up-regulation but not vice versa. Further analysis of *orf43* transcription in a Δ*orfs40/41* mutant (Δ11) [8] demonstrated that deletion of these genes, upstream of *orf43*, did not prevent the UV-induced up-regulation of *orf43* mRNA, suggesting that inducible *orf43* transcription was stimulated through a region directly in front of the *orf43* gene (Figure 2) and that this region should be investigated further. This observation is supported by previous deletion analysis where *orfs40/41* (Δ11) and ∆*orf42* (∆13) were deleted but retained the UV-inducible sensitising phenotype [8]. Analysis of the up-regulated *orfs90/91* and *orf43* mRNA decay rate post-exposure (Figure 3) revealed that *orfs90/91* mRNA levels were maximally up-regulated directly after exposure and decayed rapidly with a return to basal levels within 5 minutes post-exposure. However *orf43* mRNA levels were maximally up-regulated 7 minutes post-exposure and up-regulated levels were sustained for a longer period of time, minimally over 30 minutes (Figure 3). The observation of the rapid increase and decay of *orfs90/91* specific mRNA levels followed by a slower and longer sustained increase in *orf43* specific mRNA levels supports the hypothesis that UV irradiation acts as an inducing agent for *orfs90/91*, which subsequently up-regulates the transcription of *orf43* possibly from a site preceding the gene.

### Cytotoxic *orf43* transcription is regulated through a region directly upstream of *orf43*

**Figure 4:**
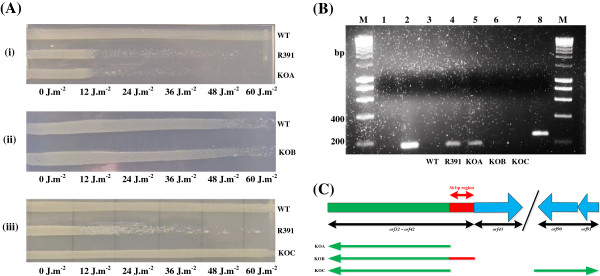
**Qualitative UV assay and mRNA analysis of *E. coli *R391 mutants KOA, KOB and KOC. (A)** AB1157 R391 mutants KOA, KOB and KOC. UV_254nm_ exposure increasing (12 J.m^-2^) from left to right. (i) From top to bottom, AB1157, AB1157 R391, AB1157 R391 KOA. (ii) AB1157, AB1157 R391 KOB. (iii) AB1157, AB1157 R391, AB1157 R391 KOC. **(B)** SYBR® Safe stained 1% (w/v) agarose gel confirming *orf43* mRNA transcription in AB1157 R391 KOA. M, Bioline Hyperladder I DNA marker; 1, AB1157 R391 RNA negative control; 2, AB1157 R391 genomic DNA positive control; 3, AB1157 *orf43* cDNA; 4, AB1157 R391 *orf43* cDNA; 5, KOA *orf43* cDNA; 6, KOB *orf43* cDNA; 7, KOC *orf43* cDNA; 8, KOB *orf20* cDNA. Primers used specific to *orf43* generated a 188 bp PCR product. Primers used for lane 8 only were specific for the kanamycin resistance gene of ICE R391, *orf20*, which generated a PCR product of 223 bp. Amplification of *orf20* specific cDNA was carried out to show KOB and KOC RNA was not degraded. Lane 1 negative control was DNase treated RNA that was not converted to cDNA. **(C)** Map of exact locations of KOA, KOB and KOC deletions on ICE R391 genome. The KOA, KOB and KOC ampicillin resistance cassettes and associated promoter were inserted into the ICE R391 genome in the reverse complement to prevent the ampicillin resistance cassette promoter inducing the transcription of *orf43* mRNA. The KOA deletion removed all possible promoters of *orf43* in front of the gene and left the last 36 bp specific to the preceding *orf42* gene. The KOB deletion removed the same region as KOA and the 36 bp region. The KOC deletion was a duplicate of KOA with an additional zeocin resistant *orfs90/91* deletion.

Based on previous observations with the Δ11 and ∆13 ICE R391 deletions, which deleted *orfs40* to most of *orf42* inclusive [8], the most likely location for an *orf43* control site would be the last 36 bp specific to *orf42* directly in front of *orf43.* Comparative bioinformatic analysis of this region and the previously documented *orfs90/91* regulated *orf4* (*jef*) [14] uncovered a short 7 bp homologous DNA sequence (5’-AGAAGAT-3’) present in front of both genes. This conserved sequence was located 77 bp upstream of *orf4* (*jef*) but directly in front of *orf43* where the last 2 base pairs of the sequence overlapped the first two base pairs of the predicted start codon of *orf43*. As no other recognisable promoter or operator region was predicted upstream of *orf43*, this 7 bp sequence may possibly represent a binding motif for the putative transcriptional enhancer (*orfs90/91*). However it is well known that transcriptional enhancer control sites can be difficult to predict [18] as they tend to be short DNA sequences lacking high sequence conservation even between enhancer types. To examine if the last 36 bp specific to *orf42* and preceding *orf43* did in fact contain a control site for *orf43* transcription, *orf43* specific mRNA expression was analysed in a number of specific deletion backgrounds spanning this putative control region [Table 1, Figure 4C]. Three directed ICE R391 deletion mutants were generated [Figure 4C] in an *E. coli* (AB1157 R391) background; the KOA deletion removed the genes *orf32* to *orf42* and placed the inserted ampicillin cassette on the reverse complement to ensure removal of all possible promoters of *orf43* transcription except for the 36 bp directly in front of *orf43*, the KOB deletion removed the genes *orf32* to *orf42* similar to KOA but additionally removed the 36 bp directly in front of *orf43* while the KOC deletion was identical to the KOA mutation, preserving the putative 36 bp control site but also contained an additional secondary zeocin resistant deletion which removed *orfs90/91*. These three deletion mutations were screened by both qualitative and quantitative UV survival assays to determine their effect on the cell-sensitising function [Figure 4A] and additionally were examined by RT-PCR to determine if *orf43* specific mRNA transcription still occurred [Figure 4B]. The KOA mutant retained the UV-inducible sensitising function [Figure 4A] and *orf43* mRNA transcription [Figure 4B], while the KOB and KOC mutations abolished the sensitising function as well as *orf43* mRNA transcription. As the KOB and KOC mutations deleted either the proposed *orfs90/91* control site or *orfs90/91* genes respectively and both of these deletions prevented cytotoxic *orf43* transcription, this analysis supported the hypothesis that *orfs90/91* encode a transcriptional enhancer complex that activates cytotoxic *orf43* transcription through the 36 bp sequence directly in front of *orf43*. In addition, cloning of *orf43* with the predicted control site in front of the gene showed that the cytotoxic function could be repressed only in cells not containing *orfs90/91* (data not shown), again supporting the hypothesis.

**Table 1:** Genotype of bacterial strains, plasmids and ICE R391 mutants used

**Strain**	**Genotype**	**Source**
AB1157	F^-^, *thr-1*, *araC14*, *leuB6*, ∆(*gpt-proA*)62, *lacY1*, *tsx-33*, *qsr’-0*, *glnV44*, *galK2*, *λ-*, *Rac-0*, *hisG4*, *rfbC1*, *mgl-51*, *rpoS396*, *rpsL31* (Str^R^), *kdgK51*, *xylA5*, *mtl-1*, *argE3*, *thi-1*	*E. coli* genetic stock centre (CGSC), Yale University, New Haven, Connecticut, USA
TOP10	F^-^, *mcrA0*, ∆(*mrr-hsdRMS-mcrBC*), φ80d*lacZ58*(M15), ∆*lacX*74, *recA1*, *araD139*, ∆(*araA-leu*)*7697*, *galU*^-^, *galK0*, *rpsL*^*-*^ (Str^R^), *endA1*, *nupG*^*-*^	Bio-Sciences, Dun Laoghaire, Dublin, Ireland
P125109	*S.* Enteritidis PT4 wild type (NCTC 13349), Nal^R^	National Collection of Type Cultures (NCTC), Salisbury, UK
**Plasmid**	**Genotype**	**Source**
pBAD33-*orf43*	Cm^R^, p15A *ori*, P_BAD_ L-arabinose inducible, *orf43*	Armshaw and Pembroke, 2013 [8]
pBAD33-*orf43*[SM12]	Cm^R^, p15A *ori*, P_BAD_ L-arabinose inducible, *orf43* containing mutation converting two leucines to prolines at a.a. position 47 and 48.	This study
pBAD33-*orf43*[SM56]	Cm^R^, p15A *ori*, P_BAD_ L-arabinose inducible, *orf43* containing mutation converting glutamine at position 115 to asparagine.	This study
pKOBEG	T_s_, P_BAD_-*gam-bet-exo cat* (Cm^R^)	Dr. P. Latour-Lambert, Institut Pasteur, 25 rue du Dr Roux, Paris, France
pUC18	Am^R^ template for deletion mutant construction	Sigma-Aldrich, Arklow, Wicklow, Ireland
pcDNA3.1^(+)^	Ze^R^ template for deletion mutant construction	Invitrogen, Bio-Sciences, Dun Laoghaire, Dublin, Ireland
**ICE**	**Genotype**	**Source**
R391	Km^R^, Hg^R^	Dr R.W. Hedges, Royal Postgraduate Medical School, London, UK
**R391 Mutant**	**Genotype**	**Source**
AB1157 R391 ∆14 (∆*orf43*)	ICE R391 *orf43* deletion strain, Am^R^, UV^-^, tra^-^	Armshaw and Pembroke, 2013 [8]
AB1157 R391 ∆26 (∆*orfs90/91*)	ICE R391 *orfs90/91* deletion strain, Am^R^, UV^-^, tra^-^	Armshaw and Pembroke, 2013 [8]
AB1157 R391 ∆11 (∆*orfs40/41*)	ICE R391 *orfs40/41* deletion strain, Am^R^, tra^-^	Armshaw and Pembroke, 2013 [8]
AB1157 R391 ∆25_Am_^R^∆14_Ze_^R^	ICE R391 *orf90 – orf94* and *orf43* deletion strain, Am^R^, Ze^R^, UV^-^, tra^-^	This study
AB1157 R391 KOA	ICE R391 *orf32 - orf42* (29575 bp – 41491 bp) deletion strain, Am^R^, tra^-^	This study
AB1157 R391 KOB	ICE R391 *orf32 - orf42* (29575 bp – 41527 bp) deletion strain, Am^R^, UV^-^, tra^-^	This study
AB1157 R391 KOC	ICE R391 *orf32 - orf42* (29575 bp – 41491 bp) and *orfs90/91* deletion strain, Am^R^, Ze^R^, UV^-^, tra^-^	This study

Str^R^ is streptomycin resistant; Cm^R^ is chloramphenicol resistant; Km^R^ is kanamycin resistant; Hg^R^ is mercury resistant; Ze^R^ is zeocin resistant; T_s_ is temperature sensitive; Nal^R^ is nalidixic acid resistant and Am^R^ is ampicillin resistant. UV^-^ is UV-inducible cell-sensitising function abolished; tra^-^ is conjugative transfer deficient mutant.

### Site-directed mutagenesis of Orf43

**Figure 5:**
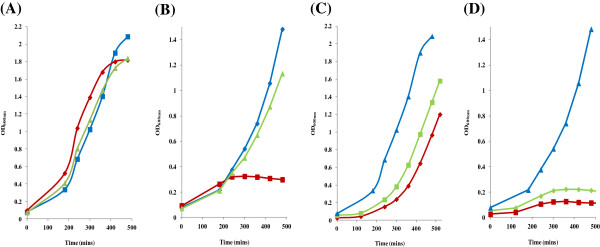
**Effect on growth rates of the pBAD33-*orf43 *SM12 and SM56 mutations in *E. coli *TOP10. (A)** Un-induced growth rates for pBAD33 (blue curve), pBAD33-*orf43* (red curve) and pBAD33-*orf43* SM12 (green curve). **(B)** Induced growth rates for pBAD33 (blue curve), pBAD33-*orf43* (red curve) and pBAD33-*orf43*[SM12] (green curve). **(C)** Un-induced growth rates for pBAD33 (blue curve), pBAD33-*orf43* (red curve) and pBAD33-*orf43* SM56 (green curve). **(D)** Induced growth rates for pBAD33 (blue curve), pBAD33-*orf43* (red curve) and pBAD33-*orf43*[SM56] (green curve). Note that the SM12 mutation in pBAD33-*orf43* caused a return to exponential growth behaviour expected with *E. coli* cells.

Bioinformatic analysis of *orf43* indicated that it belongs to a highly conserved TraV-like family of transfer proteins involved in type IV secretion systems required for conjugation [8]. Site-directed mutagenesis of pBAD33-*orf43* was carried out to convert two leucines at a.a. positions 47 and 48 to prolines in the predicted Orf43 protein (GenBank: AAM08037). Insertion of two prolines was expected to disrupt the α-helical transmembrane spanning region of Orf43 by creating a 30° bend [19]. This mutation was found to cause loss of the cytotoxic function of pBAD33-*orf43*[8] as there was no observable decline in host cell growth rates after induction of the mutant clone compared to the wild type clone [Figure 5A,B]. Since introduction of membrane disruptive mutations abolish the effect, this is suggestive that membrane association is required in addition to over-expression of the Orf43 protein for sensitisation and cytotoxicity associated with this ICE product. Other directed mutations such as conversion of glutamine at position 115 (predicted in the periplasmic region of Orf43) to asparagine (Q115 to N115) was shown to have no effect on UV sensitisation or *orf43* transcription [Figure 5C,D].

## Conclusions

### Hierarchical control of the ICE R391 UV-inducible sensitising effect

Many SXT/R391-like ICEs reduce post UV survival rates of *E. coli* host cells through the action of a *recA*-dependent process [6,20]. Mutational analysis of the ICE R391 determined that the core genes *orfs90/91* and *orf43* were required for expression of the cell-sensitising function [8] while bioinformatic analysis indicated that *orf96* likely encodes a λ cI-like repressor similar to RecA substrates in other phage systems that are cleaved following SOS induction [9]. Initial attempts to delete *orf96* proved fruitless and no deletion could be isolated. However a Δ*orf96* (Δ28) deletion [8] could be isolated in an ∆*orfs90/91* mutant background suggesting that *orf96* may control expression of *orfs90/91* which we have shown here directly control expression of *orf43*, the ultimate instigator of the cytotoxicity associated with ICE R391. The data presented here and in Armshaw and Pembroke (2013) [8] have led to the development of a model to explain the control of UV-inducible sensitisation (Figure 1). We hypothesise that UV irradiation of *E. coli* induces the host RecA protein which results in cleavage of the ICE R391 encoded product of *orf96*, the phage λ^434^ cI-like ICE repressor. We propose that cleavage of Orf96 in turn leads to expression of *orfs90/91* which in turn leads to up-regulation of *orf43* and other ICE R391 genes such as *orf4* (*jef*) [14]. We have previously demonstrated that up-regulation of *orf4* (*jef*) leads to increased ICE R391 transfer [14]. In the related ICE SXT, Beaber *et al.,* (2004) [17] demonstrated that SetR, the SXT homolog of Orf96, acted as a repressor of ICE SXT transfer and that it is bound to ICE operators that controlled *setC/D*, SXT homologs of *orfs90/91*, in a similar way to our proposal for ICE R391. They also proposed that repression was lifted by induced RecA protein cleaving the SetR repressor in a similar manner to our proposal for *orfs90/91*. The *recA* dependence for the ICE R391 UV-sensitising effect [6], the similarity to the SXT system [17], the deletion data and qRT-PCR data presented here support the model presented.

It would thus appear that UV irradiation is the instigator of the control loop leading to over expression of *orf43* which leads to cytotoxicity. Since normal levels of Orf43 are required for ICE transfer and play a key part in formation of the conjugative transfer system of ICE R391, it appears that the associated cytotoxicity is related to the induced overexpression. Evidence in support of this comes from data showing that overexpression of *orf43* from the arabinose inducible clone pBAD33-*orf43* leads directly to cytotoxicity [8]. The UV-inducible sensitising effect is conserved amongst many SXT/R391 ICE family members [6,20]. A sophisticated control system is in place to control this effect yet the exact nature and reason for conservation of such an unusual apparently ‘evolutionary negative’ effect remains to be elucidated. We are currently examining the nature of the cytotoxicity and developing theories for its function and retention.

## Methods

### Bacterial strains, elements and media

The bacterial strains, plasmids and ICE R391 deletion mutants utilised as part of this study are listed in Table 1. Strains were stored at −80°C in either Luria-Bertani (LB) broth or M9 minimal media containing 50% (v/v) glycerol. Media was supplemented with appropriate antimicrobial agents: nalidixic acid, 30 μg ml^-1^; ampicillin, 100 μg ml^-1^; chloramphenicol, 25 μg ml^-1^, kanamycin, 30 μg ml^-1^, streptomycin, 100 μg ml^-1^; mercuric chloride, 20 μg ml^-1^; zeocin, 25 μg ml^-1^ as required. For growth and analysis of strains containing pBAD33-*orf43*, M9 minimal media containing 0.4% (v/v) glycerol was used with either 0.4% (w/v) glucose or 0.02%-0.2% (w/v) L-arabinose to repress or induce gene expression respectively as previously described [8].

### Directed deletions of ICE R391 and subsequent deletion mutant screening

ICE R391 specific deletions were generated as previously described [8]. Screening of resulting ICE R391 deletion mutants for loss of cell-sensitising function by qualitative and quantitative UV survival assays were carried out as described [8]. Screening of ICE R391 deletion mutants’ conjugative transfer ability to recipient *Salmonella enterica* serotype Enteritidis strain P125109 was performed as described [21].

### Qualitative reverse transcriptase PCR

Cells were collected by centrifugation, washed twice with diethyl pyrocarbonate-treated distilled water and resuspended in 10 mM Tris, [pH8.0]. Total RNA was isolated using the Absolutely RNA Miniprep kit (Agilent Technologies) according to the manufacturer’s protocol. Absence of contaminating DNA was verified by PCR. Qualitative reverse transcriptase PCR was performed using the AccuScript High Fidelity 1^st^ Strand cDNA Synthesis Kit (Agilent Technologies) according to the manufacturer’s protocol. Resulting cDNA was analysed immediately by PCR using gene-specific primers or stored at −20°C.

### Quantitative reverse transcriptase PCR (qRT-PCR)

Quantitative UV assays were carried out as described [8]. Unirradiated and irradiated cells were collected by centrifugation and total RNA isolated as described. Absence of contaminating DNA was verified by PCR. qRT-PCR was performed using the Brilliant III Ultra-Fast SYBR Green qRT-PCR Master Mix (Agilent Technologies) according to the manufacturer’s protocol using the Stratagene Mx3000P Real Time PCR System and appropriate gene-specific PCR primers with the following temperature profile: 1 cycle at 42°C for 30 minutes to convert RNA to cDNA, 1 denaturation cycle at 95°C for 3 minutes followed by 40 cycles at 95°C for 30 seconds, 54°C for 60 seconds, 72°C for 30 seconds followed by melting curve analysis from 65°C to 95°C to determine specificity of the PCR reaction. Specificity of the PCR reaction was verified by SYBR safe staining on a 2% (w/v) agarose gel. The internal standard curve using the unirradiated RNA sample to estimate the change in target RNA quantity consisted of: undiluted RNA, a 1 in 2 dilution, a 1 in 4 dilution and a 1 in 10 dilution of unirradiated RNA. A no template negative control was also included. In addition, qRT-PCR was also carried out on the known endogenous housekeeping gene *proC* as an internal control to quantify the relative change in transcription of the gene of interest [22].

### Site-directed mutagenesis of pBAD33-*orf43*

Site-directed mutagenesis of pBAD33-*orf43*[8] was performed using specifically designed complementary mutagenic primers to linearly amplify pBAD33-*orf43* to generate a mutated nicked DNA product. Non-mutated methylated template DNA was eliminated by incubation with the *Dpn*I restriction enzyme. Mutated DNA products were then transformed into TOP10 and plated on appropriate media containing chloramphenicol, 25 μg ml^-1^. Resulting TOP10 colonies were cultured, had plasmid content extracted using the QIAprep Spin Miniprep Plasmid extraction kit from QIAGEN (West Sussex, RH10, 9NQ, UK) according to the manufacturer’s protocol and screened for the presence of pBAD33-*orf43* by restriction enzyme digestion. Mutated pBAD33-*orf43* was verified by DNA sequencing to contain the desired mutation without additional mutations. Mutated pBAD33-*orf43* was confirmed to still transcribe *orf43* specific mRNA by RT-PCR as described. Determination of the effect of induction of mutated pBAD33-*orf43* on host cell growth rate was carried out as described [8].

## Competing interests

The authors declare that they have no competing interests.

## Authors’ contributions

PA and JTP conceived and designed the study. PA did the laboratory work and analysed the data. PA and JTP wrote the manuscript. Both authors read and approved the final manuscript.
